# Impact of an eye-stroke protocol with non-mydriatic ocular imaging in an emergency department

**DOI:** 10.3389/fneur.2026.1885492

**Published:** 2026-07-30

**Authors:** Madhuri Akella, Étienne Bénard-Séguin, Andrew M. Pendley, Ghada Mohamed, Jessica G. McHenry, Daniel Adamkiewicz, Andrew Trippiedi, Lennox Xu, Stuart Duffield, Alexis Flowers, Wesley Chan, Matthew Keadey, David W. Wright, Fadi Nahab, Nancy J. Newman, Valérie Biousse

**Affiliations:** 1 Department of Ophthalmology, Emory University School of Medicine, Atlanta, GA, United States; 2 Department of Ophthalmology, University of Calgary, Calgary, AB, Canada; 3 Department of Emergency Medicine, Emory University School of Medicine, Atlanta, GA, United States; 4 Department of Neurology, Emory University School of Medicine, Atlanta, GA, United States; 5 Division of Neuro-Ophthalmology, Department of Ophthalmology and Visual Sciences, Vanderbilt University Medical Center, Nashville, TN, United States; 6 Department of Ophthalmology & Visual Sciences, Dalhousie University, Halifax, NS, Canada; 7 Department of Neurological Surgery, Emory University School of Medicine, Atlanta, GA, United States

**Keywords:** acute retinal ischemia, branch retinal artery occlusion, central retinal artery occlusion, emergency department, non-mydriatic ocular fundus photography, ophthalmic artery occlusion, optical coherence tomography

## Abstract

**Introduction:**

The diagnosis of acute central retinal artery occlusion (CRAO) and branch retinal artery occlusion (BRAO) is often delayed or missed in emergency departments (EDs) because of limited ocular funduscopic skills and lack of immediate ophthalmology access. We evaluated the clinical impact of our Eye-Stroke protocol using non-mydriatic ocular imaging (non-mydriatic fundus photography and optical coherence tomography [NMFP-OCT]) in our general ED with remote interpretation by ophthalmology on the time to diagnosis and emergent management of acute CRAO/BRAO.

**Methods:**

Prospective consecutive series of 100 acute CRAO/BRAOs seen within 1 week of vision loss between June 2023 and April 2026 with NMFP-OCT in our ED.

**Results:**

Among 100 CRAO/BRAO eyes seen within 1 week of onset, 16 (16%) had vision loss within 4.5 h, 49 (49%) between 4.5 and 24 h, 35 (35%) between 24 h and 1 week (median times from presentation to NMFP-OCT 28.5 min [IQR, 17.75–57.5 min; range, 10–330 min], 96 min [IQR, 51–180 min; range; 9–317 min], and 134 min [IQR, 96–187 min; range, 30–337 min], respectively). The diagnosis of CRAO/BRAO was made from color photographs and OCT in 77/100 eyes, from OCT only in 19/100 eyes, and 3/100 eyes had uninterpretable imaging. 7/16 eyes presenting within 4.5 h of vision loss received intravenous thrombolysis (median door-to-needle time, 58 min [IQR, 46–78.5 min; range, 41–181 min]). The majority of the patients evaluated within 4.5 h were self-referred to our ED, whereas the majority of the patients presenting later were referred by outside providers or transferred from other EDs. Stroke workup found a major cause of CRAO/BRAO in 72%, and 17/94 (18%) had concurrent cerebral infarctions on brain magnetic resonance imaging (MRI).

**Conclusion:**

Despite our Eye-Stroke protocol, facilitated by NFMP-OCT in our ED, only 16% of acute CRAO/BRAO eyes were diagnosed early enough to be considered for intravenous (IV) thrombolysis, which was administered to only 43% of those eligible. Rapid workup found a major cause of CRAO/BRAO in 72%, and 18% had concurrent cerebral infarctions on MRI. The main barrier to delayed diagnosis/care was transfer from other institutions/providers, suggesting that wide deployment of NMFP-OCT for remote diagnosis and treatment via existing telestroke networks is optimal for reducing time to diagnosis, avoiding transfers, and improving patient outcomes.

## 1 Introduction

Acute retinal ischemia, including transient and permanent ophthalmic artery, central retinal artery, and branch retinal artery occlusions (OAO, central retinal artery occlusion [CRAO], and branch retinal artery occlusion [BRAO]), is an ophthalmic emergency recognized as an ocular analog of acute cerebral ischemic stroke. These patients often have permanent severe visual loss, and a relatively high risk of recurrent retinal or cerebral ischemia, and cardiovascular events (1–6). Similar to the brain, the retina has a very short tolerance to ischemia and a time-dependent “penumbra” beyond which irreversible injury occurs. Experimental data suggest that retinal ganglion cell infarction occurs within 97–240 min in primates, although this window may be much shorter in humans, highlighting the need to administer any treatment capable of reversing retinal ischemia as early as possible (7–9).

For more than 30 years, national stroke organizations have deployed numerous strategies to alert the general population and medical providers to the need to immediately transport patients with suspected strokes to emergency departments (EDs) affiliated with a stroke center. Indeed, it is well established that immediate specialized care in a stroke center (either on-site or remotely via telestroke networks prior to transfer) is the most efficient way to optimize the chances of rapid cerebral reperfusion and to reduce short- and long-term stroke-related morbidity. However, although such stroke protocols (stroke alerts or stroke codes) have become the standard of care in all EDs, only a minority of stroke patients still reach EDs early enough to benefit from early cerebral reperfusion strategies (1, 2, 10). Acute retinal ischemic events share the same vascular risk factors and causes as cerebral ischemic strokes in the anterior circulation. The risk of concomitant and subsequent cerebral infarctions is highest within the first 2 weeks following vision loss (1, 2). Additionally, the most logical treatment strategy for acute CRAO/BRAO is to achieve reperfusion of the ischemic retina as quickly as possible. This is why an increasing number of stroke centers have implemented Eye-Stroke protocols to improve outcomes for patients with acute retinal ischemia (3, 6, 11–15).

The main obstacle to rapid diagnosis and treatment of acute retinal ischemia is the need for an ocular fundus examination looking for classic signs of CRAO/BRAO as well as for retinal arterial emboli, an assessment often delayed in general EDs where funduscopic skills are lacking and rapid access to eyecare providers is limited (12, 16). Indeed, we previously reported a dramatically low number of acute CRAO patients presenting to our academic center-affiliated large ED/stroke center (52 patients between years 2010 and 2020) (17, 18), and recent clinical trials have highlighted the difficulty recruiting CRAO patients within a few hours of vision loss (6, 19–24). Recent studies have shown that the implementation of non-mydriatic fundus photography and optical coherence tomography (NMFP-OCT) in general EDs enables ophthalmologists to remotely diagnose ocular emergencies, thereby accelerating care in the ED (25, 26) and facilitating the integration of acute retinal ischemia into established stroke pathways (11–15).

Our goal was to evaluate the impact of our Eye-Stroke protocol, which uses remote interpretation of ocular imaging obtained in the ED, on the diagnosis and management of acute retinal ischemia, similar to established brain-stroke workflows.

## 2 Methods

This was a prospective observational cohort study of consecutive patients diagnosed with acute CRAO/BRAO who received NMFP-OCT in the ED at a tertiary academic institution affiliated with a stroke center over 34 months (from June 2023 to April 2026). This study was approved by our Institutional Review Board and adhered to the Declaration of Helsinki. Informed consent was waived as our protocol followed standard-of-care practices, data were deidentified, and this study was part of a quality improvement project.

### 2.1 Eye-stroke protocol in the ED

All patients presenting to our ED with any vision complaints are triaged by a nurse who prioritizes those with acute vision loss and/or visual field changes. An order for NMFP-OCT is systematically placed for all patients with vision complaints, as previously reported (25), to facilitate triage and accelerate evaluation and management in the ED. For the subgroup of patients presenting within 6 h of vision loss, an Eye-Stroke alert is triggered, paging both on-call ophthalmology and on-call stroke neurology teams. NMFP-OCT is obtained immediately by ED staff, and the Eye-Stroke alert is confirmed by phone after an on-call ophthalmologist confirms the diagnosis of acute retinal ischemia following immediate remote interpretation of ocular imaging. The patients are not transported to the eye clinic for confirmation of the diagnosis, and in-person ophthalmology consultation with dilated funduscopic examination at the bedside or in the eye clinic is performed only after the stroke alert is activated and thrombolysis is administered (when indicated), to avoid delaying the immediate stroke evaluation. The stroke team oversees the immediate workup in the ED, which includes a standard complete stroke workup per our institution’s and AHA guidelines, in addition to C-reactive protein (CRP) and erythrocyte sedimentation rate (ESR) in patients 50 years and older (in whom giant cell arteritis must be ruled out). The stroke team determines the patient’s eligibility for off-label thrombolysis by considering the time elapsed since “last known well,” neurologic evaluation, a review of contraindications, and thorough discussions with the patient and their family.

### 2.2 Inclusion criteria

We included patients with a definite diagnosis of acute permanent retinal ischemia (OAO, CRAO, or BRAO) who presented to our adult general ED within 1 week of symptom onset and received NMFP-OCT in the ED as per our standard Eye-Stroke protocol (11, 25). Patients with transient vision loss, unclear diagnosis, incidental remote findings of acute retinal ischemia on NMFP-OCT, or delayed presentation (more than 1 week after vision loss) were excluded.

### 2.3 Patient data and treatment

We prospectively collected basic demographic information (age, sex, self-reported race/ethnicity), referral patterns, results and quality of NMFP-OCT, workup obtained, and treatments offered in the ED.

### 2.4 Referral patterns and timing of events

We collected details on the referral pattern for each patient to our institution’s ED (from an outside eyecare provider, including an optometrist or ophthalmologist, an outside hospital ED, or self-presentation directly to our ED). The distance traveled was calculated using Google Maps as the driving distance from the patient’s point of origin to our hospital. For patients presenting directly to our ED, the patient’s home zip code was used as the origin. For transferred patients, the zip code of the referring outside ED or physician’s office was used. For analysis, the selected zip code was entered into Google Maps, and the shortest driving distance to our hospital was recorded in miles. The mode of transport (by personal car or ambulance) was also determined.

We specifically recorded the time of onset of vision loss, the time of arrival in our ED, and the time of NMFP-OCT in our ED to calculate the time between vision loss onset and arrival in our ED (triage), and the time between triage and diagnosis of acute retinal ischemia on NMFP-OCT. Patients were divided into three groups based on the time of arrival at our institution’s ED: those presenting within 4.5 h of vision loss (group 1), those presenting between 4.5 and 24 h (group 2), and those presenting after 24 h (group 3). For patients in Group 1 (who were eligible per our Eye-Stroke protocol for off-label intravenous thrombolysis), we recorded the door-to-needle time for intravenous thrombolysis (when administered) and the reasons for non-treatment or treatment delays.

Medical records were reviewed to identify barriers at each step of the Eye-Stroke protocol, including factors related to the timing of patient presentation, triage, diagnostic evaluation, completion of the stroke workup, and contraindications to thrombolysis.

Results of the stroke workup and the presence of concurrent acute cerebral infarctions on head computed tomography (CT) and brain magnetic resonance imaging (MRI) were reviewed a few weeks after discharge by stroke neurologists. The final etiology of the CRAO/BRAO was categorized as arteritic (giant cell arteritis), atheroembolic (carotid and/or aortic arch), carotid dissection, cardioembolic, hypercoagulable states, other causes, or undetermined.

### 2.5 Ocular imaging studies in the ED

All patients included in this study had NMFP-OCT (without pharmacologic pupil dilation) obtained by ED staff upon presentation to our ED with acute vision loss (25). All patients received the same standard imaging protocol, including non-mydriatic 45-degree true-color fundus photographs of both eyes and OCT of the optic nerve and macula (12 × 9 mm wide-field scan), using a table-top automated non-mydriatic hybrid camera (Maestro-2, Topcon, Japan) connected to our electronic health record (EHR) system (EPIC) (25, 27). Eye tracking remained enabled throughout image acquisition and was not manually disabled. All imaging studies were immediately reviewed remotely by an on-call ophthalmologist (trained to identify signs of acute retinal ischemia using this specific camera) to confirm (or rule out) the diagnosis of acute retinal ischemia. A final interpretation of ocular imaging studies was provided by an ophthalmologist responsible for interpreting and billing all NMFP-OCTs obtained in our ED.

#### 2.5.1 Color photographs and OCT quality grading

All ocular imaging studies were subsequently reviewed retrospectively by non-blinded ophthalmologists to grade the quality of the color fundus photographs and the OCT as previously described (25, 28).

The image quality of each color fundus photograph was graded using a previously validated scale from 0 to 5, where 0 = fundus not visible, 1 = fundus minimally visible not allowing any diagnosis, 2 = fundus partially visible with poor view or no view of macula or optic nerve, 3 = fundus visualized with view of macula and optic nerve with moderate artifact or decentration, 4 = well-centered fundus image with mild artifact, and 5 = well-centered clear image without artifact (25, 28). Grades 1–2 were documented as “inadequate quality,” whereas grades 3–5 were documented as “adequate quality.”

The quality of each OCT was assigned the following quality levels: 1 = static where OCT has a static quality and minimal to no layers or features are discernible; 2 = poor where some layers of the OCT are discernible but (a) the optic nerve or the macula are not included in the field of the OCT, (b) interpretation of the optic nerve or macula is limited by poor signal, (c) there are multiple areas of “drop-out” or lost signal when scrolling through the OCT, or (d) the OCT is over-exposed and hyperreflective; 3 = moderate where all layers of the OCT are discernible with optic nerve and macula in the field of view with only one to two areas of drop-out; and 4 = good where all layers of the OCT are discernible, the optic nerve and macula are in the field of view without areas of drop-out. Grades 1–2 were documented as “inadequate quality,” whereas grades 3–4 were documented as “adequate quality” (28).

#### 2.5.2 Findings on NMFP-OCT

Color photographs and OCTs were also reviewed retrospectively by ophthalmologists to identify all visible retinal findings of acute ischemia, such as retinal whitening, cherry red spot, arteriolar box-carring (on color fundus photographs), and inner retinal layer thickening, hyper-reflectivity, paracentral acute middle maculopathy (PAMM), and loss of normal differentiation of the inner retinal layers (on macular OCT) (29–31). Presence of visible emboli was also documented. Whether each study, individually or in combination, allowed for a definite diagnosis of BRAO/CRAO was documented during the retrospective review of color photographs and OCT studies.

### 2.6 Statistical analysis

Data were expressed either as mean ± standard deviation or median with interquartile range for continuous data, as indicated, and as percentages for categorical data. Demographic variables were analyzed at the patient level, while ophthalmic outcomes were recorded at the eye level. For patients (*n* = 2) with bilateral involvement, each eye was included separately in the descriptive ophthalmic analysis. A separate subgroup analysis was performed for patients in Group 1 who were considered for thrombolysis based on time from symptom onset. Finally, we conducted a comparative analysis of the median time to presentation, patient numbers stratified by group, and temporal trends in presentation/management, both before and after implementation of our Eye-Stroke protocol with NMFP-OCT, using our consecutive data previously collected in the same ED between 2010 and 2020 (17, 18).

## 3 Results

Among 4,798 patients who underwent NMFP-OCT in our ED between June 2023 and April 2026, 116 consecutive patients (118 eyes) were diagnosed with acute retinal ischemia. A total of 98 patients (100 eyes; two patients with bilateral involvement, one simultaneous and one sequential) who presented with acute CRAO/BRAO within 1 week of vision loss were included. The median age was 69 years (range, 28–94); 49/98 patients (50%) were women and 49/98 (50%) were men. Of these, 53 (54%) patients self-identified as White people, 37 (38%) as Black people, 6 (6%) as Asian people, and 2 (2%) patients as unknown or from other ethnicity.

### 3.1 Referral patterns

A total of 47 eyes/patients of the 100 eyes/98 patients who presented with CRAOs/BRAOs included in our study were transferred to our ED “for an ocular examination” by outside hospital EDs, primarily due to the unavailability of an ophthalmologist on site. These patients traveled a median distance of 27.5 miles (IQR, 28.3 miles; range, 1.8–189 miles). A total of 36 (76.6%) patients of these 47 patients who presented with CRAOs/BRAOs were transported by ambulance, and 11 (23.4%) patients were transferred from the outside ED to our ED by personal vehicle. Of the 100 eyes/98 patients who presented with CRAOs/BRAOs, an additional 34 eyes (34%)/33 patients were referred to our ED by outside eyecare providers. They traveled a median distance of 25.5 miles (IQR, 27.85 miles; range, 2.3–231 miles). Of the 100 eyes/98 patients who presented with CRAOs/BRAOs, only 19 eyes (19%)/18 patients presented directly to our institution’s ED. They traveled a median distance of 13.4 miles (IQR, 21.35 miles; range, 0–186 miles).

### 3.2 Time to presentation and correlation with referral patterns

**Figure 1:**
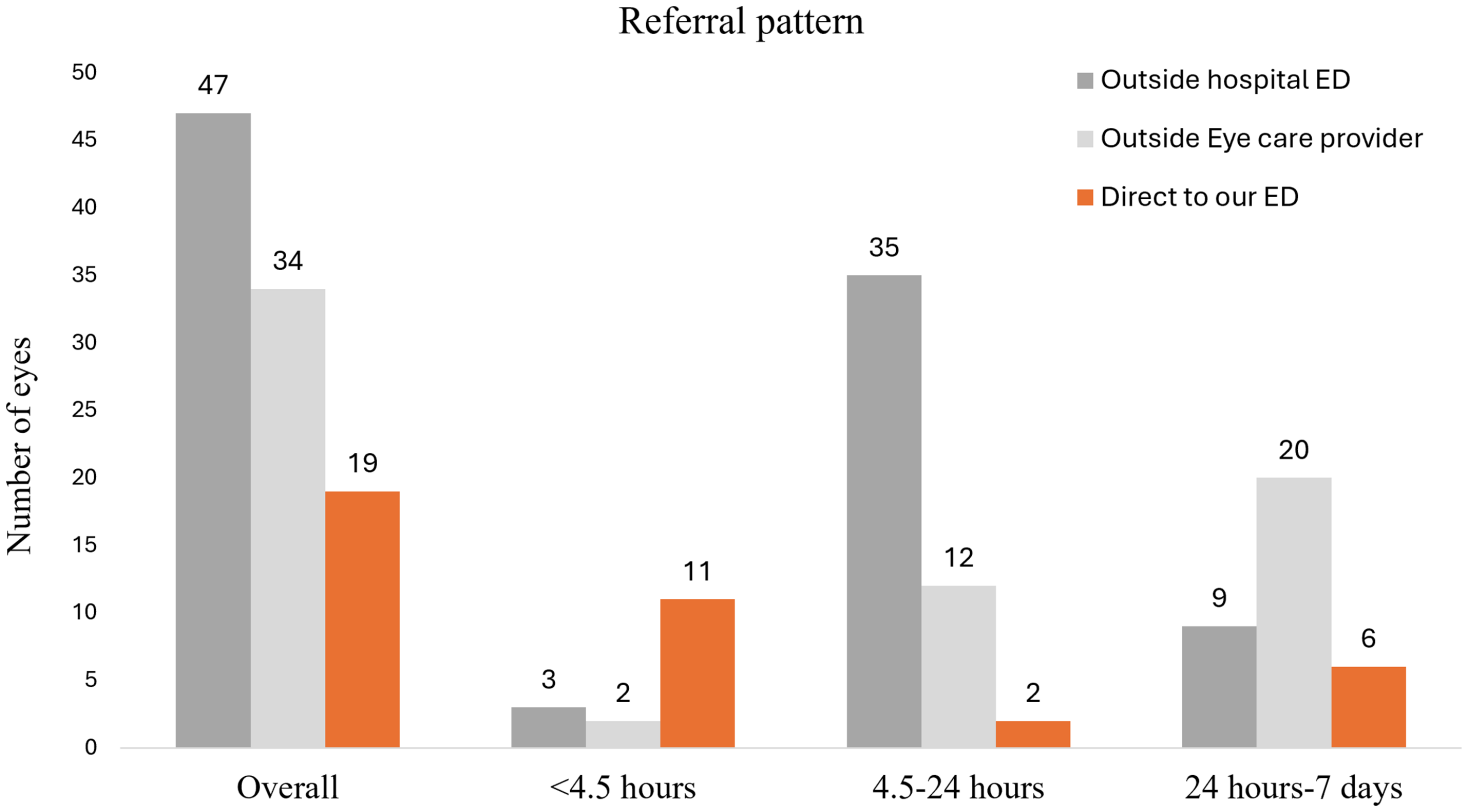
Referral patterns. The referral patterns for each eye/patient with acute retinal ischemia are shown overall and stratified by time to presentation (*x*-axis: less than 4.5 h from vision loss, 4.5–24 h from vision loss, 24 h to 7 days from vision loss; *y*-axis: number of eyes). ED: emergency department.

The overall median time from visual loss to presentation at our institution was 17.35 h (IQR, 8.06–96 h; range, 0.40–168 h). A total of 16 (16%) CRAOs/BRAOs eyes presented within 4.5 h of vision loss onset; 49 (49%) CRAOs/BRAOs eyes presented between 4.5 and 24 h, and 35 (35%) CRAOs/BRAOs eyes presented between 24 h and 1 week after vision loss (Figure 1).

Of the 16 CRAOs/BRAOs eyes who presented within 4.5 h of symptom onset (group 1), the majority, (11/16 (68.7%)), arrived directly at our ED after traveling a median distance of 15 miles (IQR, 21.2; range, 2–66.4 miles). Among the 7 CRAOs/BRAOs eyes who were treated with intravenous thrombolysis, 5 (71.4%) had presented directly to our ED after traveling a median distance of 23.1 miles (IQR, 14.35; range, 2.1–66.4 miles). A total of 47 (95.9%) of 49 CRAOs/BRAOs presenting between 4.5 and 24 h after symptom onset were either transfers from outside EDs (median distance traveled of 27.5 miles; IQR, 22.5 miles; range, 2.4–189 miles) or were referred by outside eyecare providers (median distance traveled of 42.25 miles; IQR, 43.5; range, 6.5–231 miles) (Figure 1).

### 3.3 Time to ocular imaging

**Figure 2:**
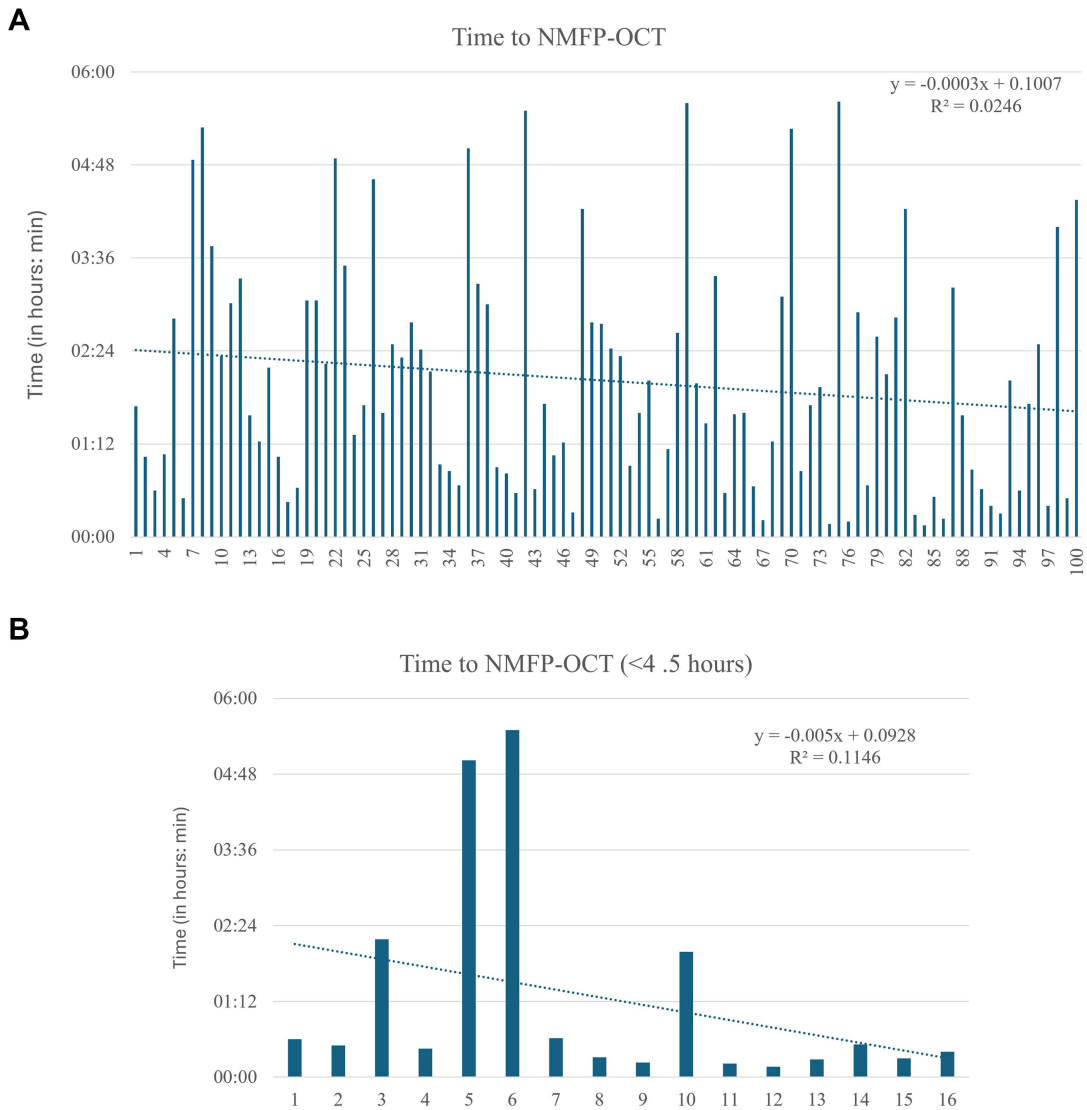
Time from presentation to our emergency department to the time of ocular imaging in the ED and to the diagnosis of acute retinal ischemia. **(A)** Graph showing the time to ocular imaging acquisition for all patients (100 eyes) organized chronologically from June 2023 to April 2026 (*x*-axis: Patient number arranged in order of being seen in our emergency department from 2023 to 2026; *y*-axis: time from arrival to ocular imaging in hours:minutes). The linear regression analysis demonstrated a slight decline in time to diagnosis/ocular imaging acquisition across sequential patient encounters (*y* = −0.0003× + 0.1007), corresponding to an approximate decrease of 26 s per patient (~43 min across the study period between 06/2023 to 04/2026), with a low coefficient of determination (*R*^2^ = 0.0246), indicating that only 2.46% of the variability in time to diagnosis is explained by the date of presentation to our ED. ED: Emergency department; NMFP-OCT: Non-mydriatic fundus photograph with optical coherence tomography of the optic nerve and macula. **(B)** Graph showing the time to diagnosis/ocular imaging acquisition for all patients presenting within 4.5 h of vision loss (16 eyes) organized chronologically from 06/2023–4/2026 (*x*-axis: Patient number arranged in order of being seen in our ED from 2023–2026; *y*-axis: time from arrival to image in hours:minutes). The linear regression analysis demonstrated a slight decline in time to diagnosis/ocular imaging acquisition across sequential patient encounters (*y* = −0.0005× + 0.0928), corresponding to an approximate decrease of 7 min per patient (~105 min across the study period between 06/2023 to 04/2026), with a low coefficient of determination (*R*^2^ = 0.1146), indicating that only 11.5% of the variability in time to diagnosis is explained by the date of presentation to our ED. ED: Emergency department; NMFP-OCT: Non-mydriatic fundus photograph with optical coherence tomography of the optic nerve and macula.

The median times between presentation and ocular imaging acquisition in the ED are shown for each group in Table 1. The time from patient arrival to NMFP-OCT in the ED decreased slightly over the 34 months studied, with an overall reduction of approximately 43 min from June 2023 to April 2026. However, this improvement was minimal on a per-patient basis (26 s) and demonstrated considerable variability (Figure 2A). In the subgroup of patients presenting within 4.5 h, this interval decreased slightly over the study period, with an overall reduction of approximately 105 min, although variability remained high (Figure 2B).

**Table 1:** Time to diagnosis of acute retinal ischemia, quality of ocular imaging, and diagnostic yield (overall and stratified by presentation time).

	Overall	Group 1 (<4.5 h)	Group 2 (4.5–24 h)	Group 3 (>1 day to 1 week)
Time to diagnose				
Median time between presentation and NMFP-OCT	102 min (IQR, 46.75–171 min; range, 9–337 min)	28.5 min (IQR, 17.75–57.5 min; range, 10–330 min)	96 min (IQR, 51–180 min; range, 9–317 min)	134 min (IQR, 95–187 min; range, 30–337 min)
Image quality				
Color photographs’ quality (adequate quality: grades 3–5)	61/100 (61%) (Mean, 3.4/5)	12/16 (75%) (Mean, 3.8/5)	31/49 (63.2%) (Mean, 3.4/5)	18/35 (51.4%) (Mean, 3.2/5)
OCT quality (adequate quality: grades 3–4)	70/100 (70%) (Mean, 3.3/4)	16/16 (100%) (Mean, 3.9/4)	33/49 (67.3%) (Mean, 3.3/4)	21/35 (60%) (Mean- 3.2/4)
Ocular imaging findings				
Retinal whitening	74/100 (74%)	8/16 (50%)	37/49 (75.5%)	29/35 (82.8%)
Cherry red spot	50/100 (50%)	5/16 (31.2%)	27/49 (55.1%)	18/35 (51.4%)
Visible embolus	13/100 (13%)	5/16 (31.2%)	4/49 (8.1%)	4/35 (11.4%)
Diagnostic yield				
Diagnosed on a color photograph	78/100 (78%)	11/16 (68.7%)	38/49 (77.5%)	29/35 (82.8%)
Diagnosed on OCT	96/100 (96%)	15/16 (93.7%)	46/49 (93.8%)	35/35 (100%)
Diagnosed on both color fundus photographs and OCT	77/100 (77%)	10/16 (62.5%)	38/49 (77.5%)	29/35 (82.8%)
Diagnosed only on color fundus photograph (missed on OCT)	1/100 (1%)	1/16 (6.2%)	0/49	0/35
Diagnosed only on OCT (missed on color fundus photograph)	19/100 (19%)	5/16 (31.2%)	8/49 (16.3%)	6/35 (17.1%)
Missed on both color fundus photograph and OCT	3/100 (3%)	0/16	3/49 (6.1%)	0/35

NMFP-OCT, non-mydriatic fundus photography and optical coherence tomography of the optic nerve and macula; IQR, interquartile range; OCT, optical coherence tomography of the optic nerve and macula.

*Post hoc* review of the two patients from Group 1 whose ocular imaging was delayed in the ED identified a camera malfunction in one patient and an inappropriate in-person ophthalmology consultation prior to ocular imaging in the other.

### 3.4 Quality of ocular imaging studies

NMPF-OCT obtained by ED staff was generally of quality good enough to allow for the remote diagnosis of acute CRAO/BRAO by ophthalmologists (Table 1). The overall mean grade of color fundus photographs was 3.4/5 and of OCTs was 3.3/4. A total of 61 of the 100 CRAOs/BRAOs (61%) had adequate quality (grades 3–5) on color fundus photographs, and 70 of 100 CRAOs/BRAOs (70%) had adequate quality (grades 3–4) on OCT (Table 1).

### 3.5 Findings on NMFP-OCT

**Figure 3:**
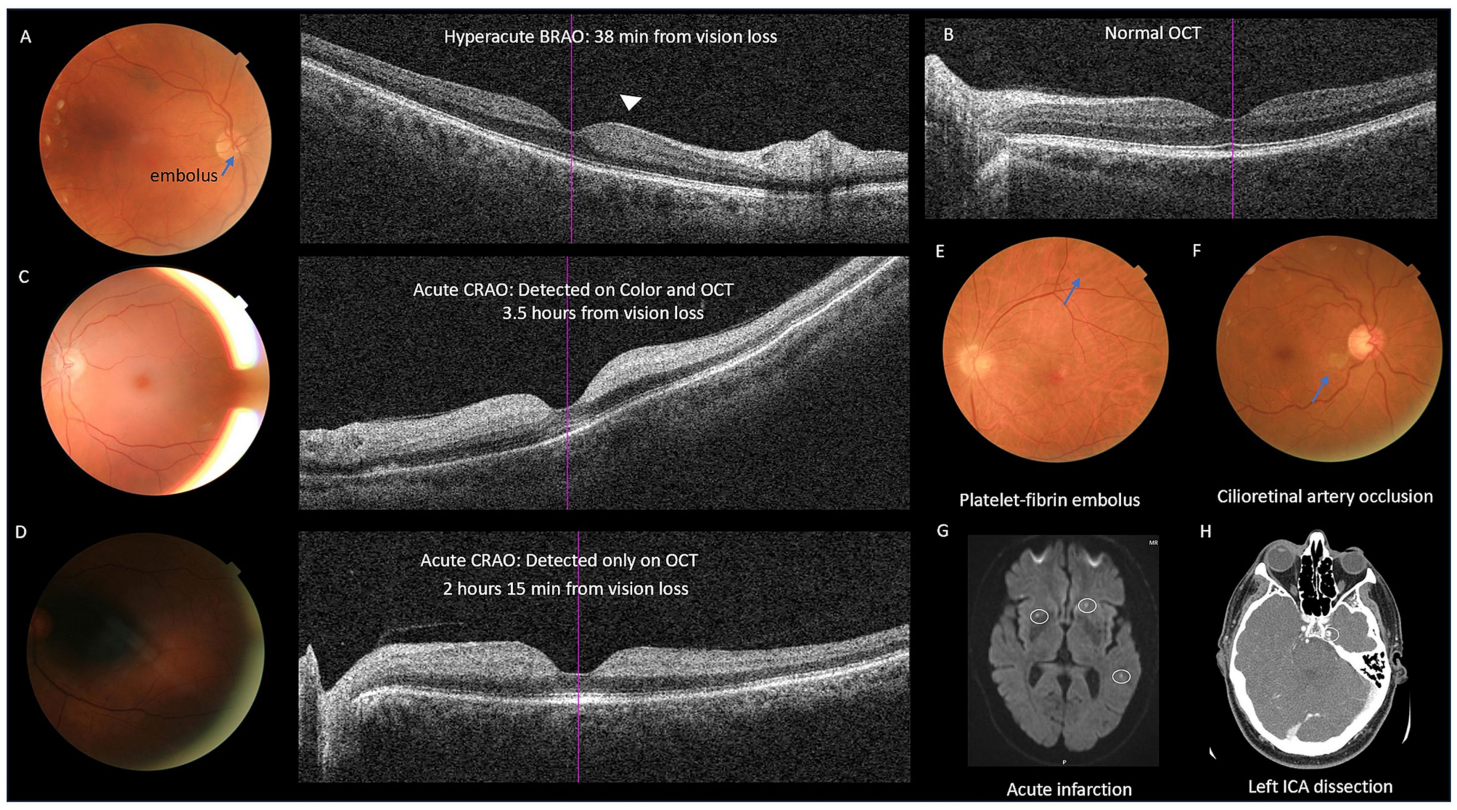
Representative cases. **(A)** Acute inferior BRAO in the right eye. Images were acquired 38 min after vision loss. There is a visible embolus along the inferior branch retinal artery (arrow) and no retinal whitening on the color photograph, but the OCT shows inner retinal hyperreflectivity compared to well-demarcated inner retinal layers on the OCT of the contralateral eye **(B)**. **(C)** CRAO with obvious retinal ischemia with retinal whitening, cherry red spot, and box-carring of vessels on color fundus photographs and inner retinal hyperreflectivity and thickening of the ischemic retina on OCT. **(D)** Poor quality fundus photograph due to poor patient fixation; however, OCT shows inner retinal hyperreflectivity. **(E)** Large visible embolus (arrow) along the superotemporal vascular arcade. **(F)** Subtle cilioretinal artery occlusion (arrow). **(G)** Diffusion-weighted MRI showing acute small asymptomatic cerebral infarction (circled). **(H)** CTA head showing left internal carotid artery dissection (circled).

Diagnostic features of acute CRAO/BRAO were present on both color fundus photography and OCT in 77/100 eyes (77%), with a cherry red spot identified in 50 eyes, retinal whitening in 74 eyes, visible retinal arterial emboli in 13 eyes, inner retinal hyperreflectivity and thickening in 85 eyes, and PAMM lesions in 11 eyes. Diagnostic features of acute CRAO/BRAO were seen exclusively on macular OCT in 19 eyes (19%), demonstrating inner retinal hyperreflectivity, thickening, or PAMM lesions (Figure 3). Three eyes with CRAO/BRAO could not be reliably diagnosed by both color photographs and OCT due to poor image quality (one eye) and absence of at least one of the two imaging studies in the affected eye (one eye with color photographs only, one eye without color photographs and OCT). In group 1 (those presenting within 4.5 h), diagnostic features were present on both color fundus photography and OCT in 10/16 eyes (62.5%), and exclusively on OCT in 5/16 eyes (31.3%). In group 2 (those presenting within 4.5 and 24 h), diagnostic features were present on both color fundus photography and OCT in 38/49 eyes (77.5%), and exclusively on OCT in 8/49 eyes (16.32%). Group 2 included the three eyes missed by both color photographs and OCT (Table 1). Ten eyes had co-existing intraocular pathologies (age-related macular degeneration, non-proliferative diabetic retinopathy, central retinal venous occlusion, myopic fundus, and choroidal nevus).

### 3.6 Thrombolysis group and barriers

Seven of 16 CRAOs/BRAOs (43.8%) presenting within 4.5 h of vision loss received intravenous thrombolysis. Median time to thrombolysis from arrival to our ED was 58 min (IQR, 46–78.5 min; range 41–181 min). Two patients did not receive thrombolysis because of contraindications. In four CRAOs/BRAOs presenting to our ED within 4.5 h of vision loss, the diagnosis of acute CRAO was delayed in the ED: in one patient, NMFP-OCT could not be obtained immediately because of malfunction of the camera; in one patient, NMFP-OCT was misinterpreted by the ophthalmologist on-call, who missed a retinal arterial embolus and subtle inner retinal hyper-reflectivity with otherwise normal-appearing retina acutely; neurologic evaluation occurred outside the treatment window in two patients who presented at 4 h after vision loss onset. The exact time of onset was debated in two patients who woke up with vision loss in one eye and for whom our stroke team did not recommend thrombolysis. One additional patient declined thrombolysis as visual acuity had started to improve spontaneously. No thrombolysis-related adverse events or safety concerns were observed in this cohort.

### 3.7 Stroke workup neuroimaging

All patients underwent a non-contrast head CT as soon as the diagnosis of CRAO/BRAO was confirmed and the Eye-Stroke alert was activated. Brain MRI was performed in 94 CRAOs/BRAOs, of whom 17 CRAOs/BRAOs (18%) had concurrent acute (15) or subacute (2) cerebral infarctions. Six CRAOs/BRAOs did not undergo brain MRI due to the following reasons: presence of a pacemaker, bullet fragment, patient refusal, leaving against medical advice, a recently negative MRI following CRAO (on the contralateral eye) performed 2 weeks prior, and a confirmed diagnosis of giant cell arteritis (GCA).

### 3.8 Stroke work-up results

Work-up in the ED/hospital showed giant cell arteritis (5/100; 5%); atherosclerotic internal carotid artery (ICA) stenosis (32/100; 32%); ICA dissection (1/100; 1%); aortic arch atheroma (6/100; 6%); cardiac source (21/100; 21%) [12 atrial arrhythmia (fibrillation/flutter), 2 valve calcification (mitral/aortic), 1 left atrial enlargement, 1 intra-atrial mass, 2 status-post cardiac valve replacement and 3 patent foramen ovale]; hypercoagulable state (6/100; 6%); invasive fungal sinusitis (1/100; 1%); and no identified cause in 28/100 (28%).

### 3.9 Comparison of the two cohorts evaluated in our ED before and after implementation of our eye-stroke protocol with NMFP-OCT

As previously published in the pre-Eye-Stroke protocol (without NMFP-OCT) period (115 months from 2010 to 2020), a total of 52 patients were identified with CRAOs/BRAOs, compared to 98 patients (100 eyes) in the post-Eye-Stroke protocol with NMFP-OCT period (34 months from June 2023 to April 2026) (17, 18). The distribution of number of eyes presenting within 4.5 h, 4.5 to 24 and 24 h to 1 week was 23.07% (12/52), 48.07% (25/52) and 28.8% (15/52), respectively, prior to 2020, compared with 16% (16/100), 49% (49/100) and 35% (35/100), respectively, between 2023 and 2026 (Table 2). There was a significant decrease in median time to diagnosis (median 3.025 h prior to NMFP-OCT compared to 28.5 min after NMFP-OCT implementation) and an increase in the proportion of eyes presenting within 4.5 h who received thrombolysis (16.6% before NMFP-OCT compared to 43.7% after NMFP-OCT) (Table 2).

**Table 2:** Distribution of eyes/patients, comparing periods before (2010–2020) and after (2023–2026) implementation of our eye-stroke protocol with NMFP-OCT in our emergency department.

	2010–2020 (17, 18) (115 months)	Current study 2023–2026 (34 months)
Number of eyes/patients presenting within 1 week	52 (patients)	98 patients/100 eyes
Median time to presentation	12 h (IQR, 4.95–48; range, 0.50–168 h)	17.35 h (IQR, 8.06–96; range, 0.40–168 h)
Median time from arrival to diagnosis of acute retinal ischemia in the ED (Group 1)	3.025 h (IQR, 1.4–5.5 h; Range, 0.45–10.15 h)	28.5 min (IQR, 17.5–57.5 min; range, 10–330 min)
Group 1: Number of eyes/patients presenting within 4.5 h of vision loss	12/52 patients (23.07%)	16/100 eyes (16%)
Group 2: Number of eyes/patients presenting within 4.5 to 24 h	25/52 patients (48.07%)	49/100 eyes (49%)
Group 3: Number of eyes/patients presenting within 24 h to 1 week	15/52 patients (28.8%)	35/100 eyes (35%)
Number of eyes/patients in Group 1 treated with thrombolysis	2/12 (16.6%)	7/16 (43.7%)

NMFP-OCT, non-mydriatic fundus photography and optical coherence tomography of the optic nerve and macula; IQR, interquartile range.

## 4 Discussion

Implementation of an acute Eye-Stroke protocol using NMFP-OCT for remote diagnosis of acute CRAO/BRAO in our ED resulted in expedited diagnosis without the need for in-person ophthalmology consultation with dilated funduscopic examination. The marked reduction in time to diagnosis from 3 h to 28 min in patients presenting within 4.5 h of vision loss highlights the protocol’s impact on accelerating the triage of acute vision loss and recognition of acute CRAO/BRAO as an emergency in our ED. The remote diagnosis of acute CRAO/BRAO on ocular imaging eliminates the delays encountered when an ophthalmologic consultation is requested by the ED, in which case pharmacologic pupil dilation is always necessary for a bedside diagnosis of acute CRAO/BRAO. Additionally, we also observed a marked increase in the number of patients with acute CRAO/BRAO presenting to our ED over the past 3 years compared with years 2010–2020 (52 patients/52 eyes over 115 months versus 98 patients/100 eyes over 34 months); however, time to presentation and distribution of presentation times remained broadly similar (17, 18). This rise may reflect growing public and healthcare provider awareness of the need to manage acute retinal ischemia as a stroke equivalent in the ED, facilitated by our own local educational interventions as well as national campaigns such as “BE-FAST” and recent publications of updated practice guidelines (2, 5, 10). This trend is not limited to our center and has been observed nationwide. However, many acute CRAO/BRAO patients continue to present too late to benefit from any potentially available very acute treatment (32). Although no causal analysis was performed, as this was not the primary aim of this study, it is also likely that the implementation of our heavily advertised Eye-Stroke protocol contributed to the large number of patients with presumed or confirmed acute CRAO/BRAO who were referred specifically to our ED by other providers.

One of the reasons for the success of our Eye-Stroke protocol, as well as other relatively similar protocols, is their incorporation into existing standard ED flow (12, 13). Since 2023, in our ED, the diagnosis of acute vision loss and potential acute retinal ischemia, based on NMFP-OCT obtained by ED staff, follows our established cerebral stroke alert pathway, with only additional laboratory evaluation to exclude giant cell arteritis (11, 25, 27, 28). The tests ordered, and practical management and treatment of acute CRAO/BRAO patients are otherwise identical to those performed for patients with suspected acute cerebral ischemic strokes.

Despite this improvement in diagnostic efficiency, substantial obstacles in pre-hospital pathways remain, including delayed patient presentation and delays related to transfer to or access to ophthalmology services (3). Indeed, only 16% of our patients presented within the treatment window for time-sensitive therapies such as intravenous thrombolysis, and nearly all the cases (96%) presenting between 4.5 and 24 h after vision loss were transfers to our ED, most commonly because of lack of on-site ophthalmology in other EDs or referral from outside eyecare providers after the diagnosis was made in their office. A study from Kaiser Permanente Northern California reported that although 51% of patients presented to one of their EDs within 4.5 h of vision loss, timely ophthalmology assessment was achieved in only one-third of cases, thereby impairing access to ultra-rapid treatments (33). Although that study was multicentric and therefore limits direct comparison with our single-center experience, both datasets highlight persistent delays between presentation and specialist evaluation in general EDs, where ophthalmologists are usually not readily available (33). Collectively, these findings support the need for broader system-level solutions, including wide deployment of NMFP-OCT and ED-based Eye-Stroke protocols. Such implementation allows for remote interpretation of ocular imaging by ophthalmologists, providing immediate, accurate diagnosis of acute CRAO/BRAO, which can then be evaluated and treated on-site (using existing telestroke services when there is no stroke center), while saving time by avoiding transfers (11–15, 25, 26).

Ocular imaging findings in CRAO are known to be time-dependent, with prior studies reporting retinal whitening in up to 81% of cases and a cherry red spot in approximately 55% (34), both of which become more obvious on funduscopic examination and color photographs with increasing time from symptom onset (5, 35). Visible emboli are generally uncommon in CRAO (<10%) and are more frequently observed in BRAO (1). In early or hyperacute presentations, funduscopic findings may be minimal or absent, as classic retinal signs of acute retinal ischemia often develop only after several h, making the diagnosis on color fundus photographs sometimes challenging. Macular OCT is particularly useful in demonstrating very early changes of retinal ischemia, such as inner retinal hyperreflectivity and thickening, as well as very subtle PAMM lesions (29–31). Our findings in patients imaged within 4.5 h of vision loss were consistent with these observations: retinal whitening in only 50%, cherry red spot in 31%, and visible emboli in 31%. Color fundus photography alone was diagnostic in 68.7% of cases, slightly less than the 84% reported in the Northern California cohort (12). Conversely, the diagnosis of very acute CRAO/BRAO was made on 93.7% of macular OCTs, highlighting the benefit of combining color photographs and OCT for the diagnosis of ocular emergencies in the ED, consistent with the experience of Lema et al. who used only OCT in their Eye-Stroke protocol (13). In our series, no hyperacute CRAO/BRAO was missed on ocular imaging when reviewing both color fundus photographs and macular OCT, regardless of the quality of imaging studies. The images were always useful despite the urgency of the diagnosis necessitating imaging without pharmacologic dilation of the pupils, the sometimes-eccentric images of the ocular fundus resulting from very poor patient vision and fixation, and the presence of co-existing retinal pathologies such as age-related macular degeneration, non-proliferative diabetic retinopathy, and central retinal vein occlusions in this older population.

All patients underwent a standard stroke workup according to our institution’s protocol and AHA/ASA guidelines. Concurrent silent cerebral infarctions were present in 18% of the 94 patients who underwent a brain MRI, and a major cause of CRAO/BRAO was found in 72% of patients, the majority of whom then required hospital admission and timely initiation of secondary prevention of stroke, reinforcing the shared vascular pathophysiology and prognosis of retinal and cerebral ischemia (1, 36). Importantly, five patients were diagnosed with giant cell arteritis, a classic cause of acute retinal ischemia, reinforcing the need to include erythrocyte sedimentation rate and C-reactive protein among the blood tests systematically obtained as part of the standard stroke workup once the diagnosis of acute retinal ischemia is made in the ED.

Although the percentage of eligible patients receiving thrombolysis in our cohort increased compared to our previous study (43% of patients presenting within 4.5 h of vision loss in our current study versus 16% of those evaluated between 2010 and 2020), the use of thrombolysis remained relatively low. While evidence for the use of thrombolysis for CRAO remains weak, current guidelines emphasize the importance of rapid diagnosis of CRAO, the need to exclude alternative ophthalmic pathology quickly, and the need to prompt identification of eligible patients within the 4.5-h therapeutic window to potentially offer this treatment off-label (2, 5). Our findings therefore support the role of structured ED-based pathways in facilitating timely assessment, even if the number of patients currently receiving treatment is limited. Furthermore, early identification of CRAO will enable timely recruitment of patients into future clinical trials and support the development of definitive vision-saving therapies, addressing a key barrier identified in recently completed randomized clinical trials (19–21).

Our study has several limitations. This was a quality improvement project conducted in a single general ED affiliated with a quaternary-care, university-based hospital, which limits generalizability to other healthcare systems with different workflows, resources, and referral patterns. We recently implemented a second camera in another ED affiliated with our institution, confirming that our model can be reproduced. Other institutions are implementing similar Eye-Stroke protocols, and a few have published results demonstrating that this model can be used in other EDs (12–15). Although our current analysis is prospective, our comparison with the pre-protocol period relies on a prior retrospective study, which may introduce inherent methodological differences between cohorts. However, the comparative data from 2010 to 2020 were collected at the same institution, in the same ED, and by the same investigator group with a similar approach to the management of acute CRAO/BRAO for more than two decades. Lastly, the few issues encountered, such as occasional camera malfunctions, poor-quality ocular imaging, delayed imaging orders with delayed diagnoses during peak ED times, and delayed neurologic evaluations, are not true limitations but rather reflect the difficulty of emergent ophthalmic evaluations in the ED, where process flows are often disrupted and unpredictable.

In conclusion, our findings demonstrate the feasibility and positive impact of an Eye-Stroke protocol using an ED-based NMFP-OCT pathway for rapid identification of acute retinal ischemia that may serve as a framework for the development and evaluation of future ultra-rapid therapies aimed at vision preservation and recovery. Emerging evidence supporting the potential for technology-driven augmentation of clinical workflow in emergency settings was highlighted in the most recent guidelines for the management of ischemic stroke (2). Integration of artificial intelligence-driven diagnostic aids and routine telemedicine networks may further enhance diagnostic efficiency, reduce unnecessary transfers, and align acute ophthalmic stroke care with existing rapid response models (37).

## Data Availability

The raw data supporting the conclusions of this article will be made available by the authors, without undue reservation.

## Author’s note

This work was presented in part at the North American Neuro-Ophthalmology Society annual meeting in Boston, Massachusetts in March 2026.
